# Iron Status Predicts Treatment Failure and Mortality in Tuberculosis Patients: A Prospective Cohort Study from Dar es Salaam, Tanzania

**DOI:** 10.1371/journal.pone.0037350

**Published:** 2012-05-11

**Authors:** Sheila Isanaka, Said Aboud, Ferdinand Mugusi, Ronald J. Bosch, Walter C. Willett, Donna Spiegelman, Christopher Duggan, Wafaie W. Fawzi

**Affiliations:** 1 Department of Epidemiology, Harvard School of Public Health, Boston, Massachusetts, United States of America; 2 Department of Nutrition, Harvard School of Public Health, Boston, Massachusetts, United States of America; 3 Department of Biostatistics, Harvard School of Public Health, Boston, Massachusetts, United States of America; 4 Departments of Global Health and Population, Harvard School of Public Health, Boston, Massachusetts, United States of America; 5 Department of Microbiology and Immunology, Muhimbili University of Health and Allied Sciences, Dar es Salaam, Tanzania; 6 Internal Medicine, Muhimbili University of Health and Allied Sciences, Dar es Salaam, Tanzania; 7 Channing Laboratory, Department of Medicine, Harvard Medical School, Boston, Massachusetts, United States of America; 8 Division of Gastroenterology and Nutrition, Children's Hospital Boston, Boston, Massachusetts, United States of America; National AIDS Research Institute, India

## Abstract

**Background:**

Experimental data suggest a role for iron in the course of tuberculosis (TB) infection, but there is limited evidence on the potential effects of iron deficiency or iron overload on the progression of TB disease in humans. The aim of the present analysis was to examine the association of iron status with the risk of TB progression and death.

**Methodology/Principal Findings:**

We analyzed plasma samples and data collected as part a randomized micronutrient supplementation trial (not including iron) among HIV-infected and HIV-uninfected TB patients in Dar es Salaam, Tanzania. We prospectively related baseline plasma ferritin concentrations from 705 subjects (362 HIV-infected and 343 HIV-uninfected) to the risk of treatment failure at one month after initiation, TB recurrence and death using binomial and Cox regression analyses. Overall, low (plasma ferritin<30 µg/L) and high (plasma ferritin>150 µg/L for women and>200 µg/L for men) iron status were seen in 9% and 48% of patients, respectively. Compared with normal levels, low plasma ferritin predicted an independent increased risk of treatment failure overall (adjusted RR = 1.95, 95% CI: 1.07 to 3.52) and of TB recurrence among HIV-infected patients (adjusted RR = 4.21, 95% CI: 1.22 to 14.55). High plasma ferritin, independent of C-reactive protein concentrations, was associated with an increased risk of overall mortality (adjusted RR = 3.02, 95% CI: 1.95 to 4.67).

**Conclusions/Significance:**

Both iron deficiency and overload exist in TB patients and may contribute to disease progression and poor clinical outcomes. Strategies to maintain normal iron status in TB patients could be helpful to reduce TB morbidity and mortality.

## Introduction

With the expansion of high-quality directly observed treatment short course programs over the last 15 years, important progress has been made in reducing the global burden of tuberculosis (TB). TB disease, however, still carries an appreciable mortality, representing the world's second leading cause of death from a single infectious agent [1]. Variability in disease progression, with approximately 10% of TB-infected individuals developing clinical disease in the absence of immunosuppression [2], suggests that individual factors may play a role in the response to infection.

Micronutrient status, an important contributor to immune function and cytokine kinetics, has been increasingly suggested to play a role in the individual response to TB. The potential role of iron in TB was first reported in 1872 when a French physician noted a greater risk of relapse among recovering TB patients given iron-rich supplements [3]. Considerable experimental evidence has since accumulated demonstrating the contribution of iron availability to mycobacterial growth in serum, cells and mice [4], [5], [6], [7].

Nevertheless, there are few reports on host iron status at the time of TB diagnosis, and limited epidemiological research has been conducted on the potential effects of iron status on the progression of TB disease. A small number of studies have shown elevated iron levels to be associated with an increased risk of TB infection and death from TB [8], [9], [10]. Iron deficiency has been associated with impaired immune function in in vitro and in vivo models [11], [12], but evidence on the role of iron deficiency in the context of human TB disease is limited.

To better understand the role of iron status in the progression of TB, we used data collected from a micronutrient supplementation trial conducted among adult TB patients in Dar es Salaam, Tanzania to assess the association of iron status with clinical outcomes in TB. We used plasma levels of ferritin, an iron storage protein that circulates in quantities proportional to the amount of iron storage, as an indicator of iron status and related high and low plasma ferritin at baseline with the risk of treatment failure, TB recurrence and mortality.

**Table 1:** Socio-demographic and clinical characteristics of 705 tuberculosis patients at baseline by level of iron status.

	Baseline iron status^a^			
	Low	Normal	High	*P* b
	n (%)	n (%)	n (%)	
All	65 (9)	302 (43)	338 (48)	
*Socio-demographic characteristics*				
Female	40 (62)	108 (36)	80 (24)	<0.0001
Median age, y (IQR)	27 (23, 31)	30 (24, 36)	32 (27, 39)	<0.0001
Highest educational attainment				0.46
None	7 (11)	37 (12)	26 (8)	
Incomplete primary	7 (11)	34 (11)	34 (10)	
Incomplete secondary	46 (71)	202 (67)	236 (70)	
Secondary or higher	5 (8)	29 (10)	42 (12)	
Median household size^c^ (IQR)	4 (3, 7)	4 (2, 6)	4 (2, 6)	0.54
Median number of household assets (IQR)	1 (0, 2)	1 (0, 2)	2 (0, 3)	0.0007
Median amount spent on food (IQR)^d^	375 (250, 667)	500 (286, 750)	500 (286, 750)	0.43
*Clinical characteristics*				
Hemoglobin <11 g/dL	46 (71)	174 (58)	214 (63)	0.09
C-reactive protein >10 mg/L	30 (46)	208 (69)	291 (86)	<0.0001
HIV-infected	29 (45)	138 (46)	195 (58)	0.005
History of tuberculosis disease	45 (69)	206 (68)	214 (64)	0.39
Number of colonies in AFB culture				0.32
None	4 (6)	18 (6)	18 (5)	
1–100	38 (58)	167 (55)	165 (49)	
>100	23 (35)	116 (39)	155 (46)	
Karnofsky score <70%	2 (3)	30 (10)	46 (14)	0.03
Median BMI (kg/m^2^, IQR)	19 (18, 22)	19 (18, 21)	19 (17, 21)	0.05
Median CD4 T cell count (cells/uL, IQR)	546 (374, 832)	530 (316, 752)	473 (214, 679)	0.005
WHO HIV clinical stage^e^				0.79
Stage 3	18 (90)	93 (92)	137 (90)	
Stage 4	2 (10)	8 (8)	16 (10)	
HIV RNA>50,000 copies/mL^e^	11 (42)	62 (56)	91 (55)	0.41

Values are n (%), unless otherwise stated. Totals may be less than 705 due to missing values.

Abbreviations used: IQR, inter-quartile range, AFB, acid-fast bacilli, BMI, body mass index, WHO, World Health Organization.

a Baseline iron status was categorized as low: plasma ferritin <30 µg/L; normal: plasma ferritin 30 to ≤150 µg/L for women and 30 to ≤200 µg/L for men; and high: plasma ferritin >150 µg/L for women and >200 µg/L for men.

b *P* value is from the χ^2^ test for proportions and the Kruskal-Wallis test for continuous measures.

c Household size was defined as the number of people eating in the household.

d Amount spent on food is in Tanzanian shillings per person per day. At the time of the start of the study in 2000, the mean exchange rate was 1 USD = 799 Tanzanian shillings.

e WHO clinical stage and HIV RNA assessed only in HIV-infected patients, n = 362.

## Methods

### Study population

Between April 2000 and April 2005, 887 adults with pulmonary TB (471 HIV-infected and 416 HIV-uninfected) were enrolled in a randomized, placebo-controlled trial to examine the effect of micronutrient supplementation on sputum conversion (defined as the finding of an acid-fast bacilli [AFB] negative sputum culture) at one month, TB recurrence, and mortality. Details of the trial have been published elsewhere (ClinicalTrials.gov identifier NCT00197704) [13]. Patients were randomized, stratified by HIV status, to receive a daily oral dose of multiple micronutrients (not including iron) or placebo; all patients received an eight-month short course of anti-TB treatment in accordance with the Tanzania National Tuberculosis and Leprosy Program guidelines.

**Table 2:** Association of baseline iron status with treatment failure at one month after initiation.

Iron status^a^	# Events/	Unadjusted Model	Multivariate Model^b^
	No. at risk	Relative risk (95% CI)	Relative risk (95% CI)
**Overall**			
Low	12/50	1.70 (0.94, 3.07)	1.95 (1.07, 3.52)
Normal	30/212	1.00	1.00
High	52/251	1.46 (0.97, 2.21)	1.54 (1.00, 2.39)
**HIV-infected patients**			
Low	5/22	1.82 (0.71, 4.63)	2.21 (0.96, 5.10)
Normal	12/96	1.00	1.00
High	21/141	1.19 (0.62, 2.31)	1.26 (0.63, 2.53)
**HIV-uninfected patients**			
Low	7/28	1.61 (0.75, 3.48)	1.82 (0.82, 4.05)
Normal	18/116	1.00	1.00
High	31/110	1.82 (1.08, 3.05)	1.75 (1.04, 2.95)

a Baseline iron status was categorized as low: plasma ferritin <30 µg/L; normal: plasma ferritin 30 to ≤150 µg/L for women and 30 to ≤200 µg/L for men; and high: plasma ferritin >150 µg/L for women and >200 µg/L for men.

b Adjusted risk ratio from a log-binomial regression model adjusting for baseline covariates including sex, age (years), money spent on food per person per day (<500, ≥500 TSH), number of colonies in AFB culture, Karnofsky score (<70%, ≥70%), BMI (kg/m^2^), history of TB disease (yes/no), HIV infection status, CD4 T cell count (cells/uL) and log HIV RNA (copies/mL), trial regimen and C-reactive protein (mg/L).

*P* value, test for interaction by HIV infection status = 0.55.

### Ethics statement

The study was approved by the institutional review boards of Muhimbili University of Health and Allied Sciences, the Tanzanian National AIDS Control Program of the Tanzanian Ministry of Health, and Harvard School of Public Health. Written informed consent was obtained from all patients.

**Table 3:** Association of baseline iron status with tuberculosis recurrence.

Iron status^a^	# Events/	Unadjusted Model	Multivariate Model^b^
	Person-mo	Relative risk (95% CI)	Relative risk (95% CI)
**Overall**			
Low	6/634	0.91 (0.38, 2.18)	1.05 (0.42, 2.63)
Normal	34/3,819	1.00	1.00
High	34/3,398	1.04 (0.65, 1.67)	1.39 (0.80, 2.39)
**HIV-infected patients**			
Low	5/275	2.52 (0.85, 7.54)	4.21 (1.22, 14.55)
Normal	9/1,557	1.00	1.00
High	17/1,770	1.53 (0.68, 3.44)	2.43 (0.93, 6.34)
**HIV-uninfected patients**			
Low	1/358	0.23 (0.03, 1.71)	0.23 (0.03, 1.72)
Normal	25/2,262	1.00	1.00
High	17/1,628	0.91 (0.49, 1.68)	1.09 (0.56, 2.10)

a Baseline iron status was categorized as low: plasma ferritin <30 µg/L; normal: plasma ferritin 30 to ≤150 µg/L for women and 30 to ≤200 µg/L for men; and high: plasma ferritin >150 µg/L for women and >200 µg/L for men.

b Adjusted relative risk from a proportional hazards model adjusting for baseline covariates including sex, age (years), money spent on food per person per day (<500, ≥500 TSH), number of colonies in AFB culture, Karnofsky score (<70%, ≥70%), BMI (kg/m^2^), history of TB disease (yes/no), HIV infection status, CD4 T cell count (cells/uL) and log HIV RNA (copies/mL), trial regimen and C-reactive protein (mg/L).

*P* value, test for interaction by HIV infection status = 0.02.

### Follow-up

Monthly follow-up was conducted by research nurses at the study clinics and included anthropometric assessment and recording of any illness in the previous month. Patients were followed until death, loss-to-follow-up or study closure in August 2005. Sputum specimens for AFB smear and culture and blood specimens for the measurement of hemoglobin and T-lymphocyte subsets were collected at baseline; 1, 2, 5, 8, and 12 months after treatment initiation; and every 6 months thereafter. Blood collected at baseline, 2, 8 and 14 months among HIV-infected patients was also measured for HIV RNA levels. HIV disease stage was assessed every 3 months at physician visits according to the WHO staging system for HIV disease.

**Table 4:** Association of baseline iron status with mortality and HIV disease progression from WHO stage 3 to stage 4.

Iron status^a^	# Events/	Unadjusted Model	Multivariate Model^b^
	Person-mo	Relative risk (95% CI)	Relative risk (95% CI)
**Mortality**			
Overall			
Low	7/2,801	0.97 (0.43, 2.18)	0.86 (0.36, 2.05)
Normal	34/13,345	1.00	1.00
High	83/12,375	2.54 (1.70, 3.79)	3.02 (1.95, 4.67)
HIV-infected patients			
Low	6/990	0.85 (0.36, 2.04)	0.82 (0.33, 2.05)
Normal	33/4,625	1.00	1.00
High	72/5,409	1.86 (1.23, 2.81)	2.70 (1.72, 4.24)
HIV-uninfected patients			
Low	1/1,812	4.71 (0.30, 75.24)	2.50 (0.13, 47.32)
Normal	1/8,720	1.00	1.00
High	11/6,967	13.52 (1.75, 104.49)	12.81 (1.63, 101.02)
**HIV disease progression**			
Low	9/522	1.28 (0.61, 2.71)	1.70 (0.71, 4.08)
Normal	30/2,211	1.00	1.00
High	40/2,471	1.16 (0.72, 1.87)	1.25 (0.73, 2.14)
**HIV disease progression or death**			
Low	9/612	0.97 (0.47, 1.99)	0.82 (0.37, 1.84)
Normal	41/2,563	1.00	1.00
High	71/3,199	1.36 (0.93, 2.00)	1.34 (0.87, 2.06)

a Baseline iron status was categorized as low: plasma ferritin <30 µg/L; normal: plasma ferritin 30 to ≤150 µg/L for women and 30 to ≤200 µg/L for men; and high: plasma ferritin >150 µg/L for women and >200 µg/L for men.

b Adjusted relative risk from a proportional hazards model adjusting for baseline covariates including sex, age (years), money spent on food per person per day (<500, ≥500 TSH), number of colonies in AFB culture, Karnofsky score (<70%, ≥70%), BMI (kg/m^2^), history of TB disease (yes/no), HIV infection status, CD4 T cell count (cells/uL) and log HIV RNA (copies/mL), trial regimen and C-reactive protein (mg/L).

*P* value, test for interaction by HIV infection status for mortality endpoint = 0.22.

### Laboratory procedures

Sputum specimens were prepared and stained using the Ziehl-Neelsen technique and examined for AFB using direct microscopy by trained laboratory technicians. AFB-positive specimens were cultured for *M. tuberculosis* using Lowenstein Jensen medium and examined weekly for a maximum of 8 weeks. Plasma HIV RNA levels in HIV-infected patients was determined using the Amplicor HIV-1 Monitor assay version 1.5 (Roche Diagnostic Systems, Branchburg, NJ). After study closure, in 2010 we drew on plasma specimens stored at −80°C since collection to measure plasma ferritin and C-reactive protein (CRP) using a particle-enhanced immunoturbidimetric assay (Roche Diagnostics GmbH, Mannheim, Germany) in the Roche Cobas Integra 400 Plus analyzer.

### Classification of iron status

We used plasma ferritin concentration as an indicator of iron status. Ferritin is considered a reliable measure of iron status in many settings, but as a positive acute phase reactant, concentrations can increase during inflammation or infection even if iron status is low. In the absence of an acute phase response, ferritin concentrations <12 to 15 µg/L are indicative of depleted iron stores [14]; however, in the context of TB infection, a higher threshold of <30 µg/L has been suggested to increase sensitivity of this indicator [15]. Ferritin concentrations consistent with high iron status have varied in the literature, but it has been suggested that levels of >150 µg/L for women and >200 µg/L for men are indicative of iron overload [16]. In this analysis, we defined three levels of iron status by baseline plasma ferritin concentration as follows: low (plasma ferritin <30 µg/L); normal (plasma ferritin 30 to ≤150 µg/L for women and 30 to ≤200 µg/L for men); and high (plasma ferritin >150 µg/L for women and >200 µg/L for men).

### Statistical analysis

We compared subject characteristics at baseline by levels of plasma ferritin using the χ^2^ test for categorical variables and the Kruskal-Wallis test for continuous variables. We considered two TB-related endpoints: treatment failure at one month, defined as an AFB-positive sputum culture at one month from treatment initiation, and TB recurrence, defined as a positive culture after one month among those who were culture negative by one month after treatment initiation. Recurrence included both endogenous re-activation and exogenous re-infection, as the two could not be distinguished in the current study. Log-binomial regression was used to assess the associations of baseline plasma ferritin with treatment failure [17]. Cox proportional hazards regression was used to assess the associations of baseline plasma ferritin with time to TB recurrence and death. Among HIV-infected patients, we also fit Cox proportional hazards regression models to examine the relationship of plasma ferritin at baseline with time to HIV disease progression from stage 3 to stage 4 and the combined endpoint of death or HIV disease progression.

We adjusted for potential baseline confounders, including sex, age (years), money spent on food (<500, ≥500 Tanzanian shillings per person per day, equivalent to US $0.63 at the time of the start of the study), number of colonies in AFB culture, Karnfosky score (<70%, ≥70%), history of previous TB disease (yes/no), HIV infection status (infected/uninfected), BMI (kg/m^2^), CD4 T cell count (cells/uL), log HIV RNA (copies/mL), as well as randomized trial regimen (micronutrient treatment/placebo). Given the known influence of inflammation on plasma ferritin concentrations, we additionally adjusted for CRP (mg/L), a reliable indicator of inflammation, in all multivariate models. Continuous covariates modeled using restricted cubic splines to allow for non-linearity [18], [19]. When non-linear associations were found, the corresponding spline terms chosen through stepwise selection were included in the model.

We assessed the associations of baseline plasma ferritin with treatment failure, TB recurrence, and mortality in the overall cohort and according to HIV infection status. Because plasma ferritin concentrations increase during the acute phase response and high levels may not represent high iron status in the presence of inflammation, we also assessed the association of high plasma ferritin among subjects with low levels of CRP (CRP≤10 mg/L) when a significant association was observed overall. Analyses were performed using SAS version 9.1 (SAS Institute Inc, Cary, NC). *P* values were two-sided and considered statistically significant at *P*<0.05.

## Results

Among the 887 TB patients included in the parent trial, 705 (79%) had a plasma ferritin measurement at baseline and were eligible for analysis. Overall, 9% of patients had low plasma ferritin and 48% had high levels. The baseline socio-demographic and clinical characteristics of patients according to level of plasma ferritin are shown in **Table 1**. Patients with high plasma ferritin were more likely to be female and older but were similar to patients with low and normal plasma ferritin with respect to other socio-demographic characteristics, including education, household size, and money spent on food. The prevalence of high CRP concentrations increased and CD4 T cell counts decreased with increasing plasma ferritin concentrations, but other clinical characteristics (i.e. history of previous TB disease, mean hemoglobin, and BMI) did not significantly differ by plasma ferritin level.

Information of sputum culture status at one month after treatment initiation was available for 513 (73%) patients with a plasma ferritin measurement at baseline, of whom 94 (18%) were culture positive at one month. Compared with patients with normal plasma ferritin, the risk of treatment failure at one month was independently increased among those with low levels of plasma ferritin (**Table 2**, adjusted RR = 1.95, 95% confidence interval (CI): 1.07 to 3.52). We found no evidence for an association of the risk of treatment failure with high levels of plasma ferritin.

We next assessed the association between plasma ferritin and TB recurrence. Of the 394 patients with a negative culture at one month, 74 (19%) had a subsequent positive culture. The risk of TB recurrence was modified by HIV infection status (*P* for interaction = 0.02). Neither low nor high plasma ferritin significantly predicted TB recurrence among HIV-uninfected patients (**Table 3**). In contrast, among HIV-infected patients and after adjustment for potential confounders including CRP, low plasma ferritin was associated with an independent 4.2-fold increase (95% CI: 1.22 to 14.55) in the risk of TB recurrence.

With a median duration of 47 months of follow-up, we documented 124 deaths, 111 among HIV-infected patients and 13 among HIV-uninfected patients. The relative risks of mortality by level of plasma ferritin are shown in **Table 4**. After adjustment for potential confounders including CRP, high plasma ferritin was associated with an independent 3-fold increase in the risk of death overall (95% CI: 1.95 to 4.67). Because plasma ferritin concentrations increase during the acute phase response and high levels may not represent high iron status in the presence of inflammation, we also assessed the association of high plasma ferritin with mortality among subjects without evidence of inflammation. Among subjects with CRP≤10 mg/L (*n* = 176), high plasma ferritin remained a strong, significant predictor of the risk of death (adjusted RR = 5.05, 95% CI: 2.42 to 10.54).

## Discussion

Our results from a large prospective study of TB patients provide additional evidence for the role of host iron status in TB disease progression. We found that low plasma ferritin was associated with an increased risk of treatment failure at one month after treatment initiation overall and of TB recurrence among HIV-infected patients. High plasma ferritin, independent of the acute phase response, was associated with an increased risk of mortality.

We found a somewhat small proportion (9%) of patients in this population to be iron deficient, as defined by plasma ferritin. Our results are consistent with the low prevalence of iron deficiency reported in similar cohorts of TB patients [20], 8.5% of women and 2.9% of men with serum ferritin <24 µg/L in rural Tanzania [21] and 0% of patients with serum ferritin <14 µg/L in South Africa [22]). Many patients in this study, however, showed evidence of an acute phase response reflected by elevated CRP concentrations. Since plasma ferritin concentrations increase during the acute phase response, it is possible that the true burden of iron deficiency is under-estimated when assessed using ferritin concentrations. In a previous analysis from this cohort, 53% of patients at baseline were found to be iron deficient as defined by mean corpuscular volume <80 fl [23].

We found strong, positive associations of low iron status with the risk of treatment failure at one month overall and of TB recurrence among HIV-infected patients. These results are based on a relatively small number of events and should be confirmed in larger cohorts. They are, however, consistent with the hypothesis that iron deficiency may impair immune function [11], [12] and lead to increased susceptibility or progression of TB disease. Experimental evidence suggests that iron deficiency may compromise cell-mediated immunity by decreasing lymphocyte activation and proliferation and reducing macrophage bactericidal activity [24], [25]. Iron deficiency has also been shown experimentally to alter the Th1/Th2 cytokine balance, promoting a strong Th2 response that has been associated with clinical TB disease [26], [27], [28]. Evidence underlying the potential effect of iron deficiency in TB is experimental and requires extrapolation from in vitro and animal models to humans, but we suggest that the potential magnitude of increased risk and plausible mechanisms support the need for further study. Future studies on the potential impact of host iron deficiency in TB progression should aim to use multiple markers of iron deficiency, including those less influenced by the acute phase response such as soluble transferrin receptor.

We found that elevated ferritin levels were strongly associated with an increased risk in mortality. High splenic iron was associated with an increased risk of death from TB in an autopsy series from South Africa [10]. High dietary iron [9] and bone marrow macrophage iron [8] have also been associated with increased odds of TB. One hypothesized mechanism linking high iron status to TB disease susceptibility and progression is that increased iron availability stimulates microbial growth. Evidence from in vitro and animal models suggests that iron loading can increase mycobacterium replication [4], [5], [6]. Laboratory studies also indicate that iron loading of macrophages, which are critical to the body's defense against TB, impairs the ability of these cells to inhibit the growth of intracellular mycobacteria [29], [30]. Recent evidence from TB patients in rural Zimbabwe also suggests that increased dietary iron may attenuate the Th1 immune response [31], which is thought to contribute to the control of TB infection.

Because ferritin is a positive acute phase reactant, it can be argued that elevated ferritin concentrations could reflect either increased iron status or inflammation in the setting of normal iron status. We examined the association of high plasma ferritin levels independent of inflammation in two ways: multivariate adjustment for CRP and restriction to low levels of CRP. We would expect both methods to attenuate the observed associations for high plasma ferritin if due to inflammation and not iron status per se. After multivariate adjustment and restriction, however, we found the observed associations were not attenuated but rather strengthened. While residual confounding remains a possibility, our finding that high plasma ferritin remained significantly associated with poor clinical outcomes after accounting for CRP concentrations provides support for the clinical importance of iron overload in TB progression.

This study has several limitations. First, the interpretation of plasma ferritin concentrations is complicated during conditions eliciting an acute phase response. We used multivariate adjustment and stratification by CRP concentration to help better relate high plasma ferritin to iron status but are unable to distinguish other pathways that may contribute to elevated iron status, such as high dietary intake of iron and factors associated with its bioavailability, or genetic polymorphisms. Second, we did not have data on several predictors of iron status and known risk factors for TB infection, including smoking, alcohol history and diabetes. The prevalence of these factors is not likely high in this population [32], [33], but we cannot rule out the contribution of confounding to the observed associations. Third, it has been shown that antiretroviral treatment for HIV may modulate iron status [34], but at the time of this study, antiretroviral treatment was not available to the majority of HIV-infected individuals in Tanzania. Analysis within this cohort allows us to better understand any potential adverse effect of iron status on the natural history of TB-HIV co-infection but may limit the generalizability of these findings as antiretroviral therapy becomes more accessible.

Anemia is highly prevalent among TB patients [20], [35] and is often treated with iron supplementation, irrespective of etiology. Concerns have been raised about the safety of iron supplementation and iron overload in the context of infectious diseases, but the available evidence is conflicting [10], [36], [37], [38]. While our results are not conclusive, they are in agreement with the hypotheses that iron imbalance at both ends of the continuum exist in TB patients and may contribute to disease progression and poor clinical outcomes.

These results have practical clinical and programmatic implications. In contrast to liver biopsy and bone marrow aspiration, the gold standards for the diagnosis of iron overload and deficiency, plasma ferritin is relatively easy and inexpensive to measure. Thus where extensive clinical investigations are impractical, we show that routine screening for ferritin may be used to identify those at risk for poor clinical outcomes. The close monitoring of patients with abnormal ferritin values could be used to reduce TB morbidity and mortality. Programs recommending iron supplementation for the treatment of anemia in regions with a high burden of TB may also consider targeting the use of supplemental iron to those patients with evidence of iron deficiency. Further study in TB-infected populations is needed to better understand the potential benefit of treating anemia with iron supplementation and risk of increasing iron status to potentially hazardous levels.

In conclusion, we found both low and high iron status to be associated with poor treatment outcomes and mortality in TB. The plausible mechanisms, consistency with previous results, and the magnitude of the observed associations strongly suggest that the potential risks associated with iron imbalance should be investigated further. Continued advances in our understanding of how iron status may modulate the pathogenesis and progression of TB could be of critical importance to improving the clinical management of TB patients.
